# Pancytopenia in children: A 6-year spectrum of patients admitted to Pediatric Department of Rehman Medical Institute, Peshawar

**DOI:** 10.12669/pjms.295.3865

**Published:** 2013

**Authors:** Anwar Zeb Jan, Bakhtyar Zahid, Samreen Ahmad, Zahid Gul

**Affiliations:** 1 Dr. Anwar Zeb Jan, MRCP, Pediatric Department Rehman Medical Institute, Peshawar, Pakistan.; 2 Dr. Bakhtyar Zahid, MRCPCH, Pediatric Department Rehman Medical Institute, Peshawar, Pakistan.; 3 Dr. Samreen Ahmad, FCPS, Pediatric Department Rehman Medical Institute, Peshawar, Pakistan.; 4 Dr. Zahid Gul, MCPS (Trainee), Pediatric Department Rehman Medical Institute, Peshawar, Pakistan.

**Keywords:** Anemia, Bone Marrow, Megaloblastic, Pancytopenia

## Abstract

***Objective:*** To determine the various spectrum of pancytopenia with its frequency on the basis of bone marrow examination in children from 6 months to 14 years.

***Methods: ***A retrospective descriptive study was carried out at Department of Pediatric Rehman Medical Institute Peshawar from January 2006 to December 2012. A total of 205 patient’s age between 6 months and 14 years, fulfilling the inclusion and exclusion criteria were included in the study. Complete blood count, peripheral smear, bone marrow examination and Serum vitamin B12 level was done in all the cases.

***Results:*** Out of 14642 patients admitted to the Pediatric department during the study period, 205 (1.4%) patients were pancytopenic on their peripheral blood smear. Male outnumbered female with a ratio of 1.8:1. 42.5% of the patients were in the age group of 1 month to 5 years. Common etiological pattern identified were Aplastic anemia 58(28.3%), Hematological malignancies 49 (23.9%), megaloblastic anemia 40 (19.5%), idiopathic thrombocytopenic purpura 16 (7.8%), iron deficiency anemia 9 (4.4%), hemolytic anemia 7 (3.41%), Visceral leishmaniasis 6 (2.93%), hypersplenism 5 (2.44%), malaria 5 (2.44%), anemia of chronic disorder 4 (1.95%), Myelodisplastic syndrome 3 (1.46%), Niemen pick disease 2 (0.97%) and Gaucher disease in 1(0.49%). Common clinical presentations were fever, pallor, body aches, petechial hemorrhages and epistaxis.

***Conclusion:*** Pancytopenia is one of the importance occurrences in pediatric patients. Acute leukemia and bone marrow failure are the most common causes yet megaloblastic anemia and infections are the treatable and reversible causes of pancytopenia.

## INTRODUCTION

Pancytopenia is a malady in which there is lessening of all the three cellular elements of blood; prevailing when the hemoglobin (Hb) <10g%, absolute neutrophil count (ANC) <1.5*109/L, platelet count <100*109/L. The pancytopenia was labeled as severe if patient had two or more of the following: Hb <7 gm%, ANC <0.5*109/L, and platelet count <20*109/L.^1^ In pancytopenia the marrow is customarily hypocellular as a result of primary production defects, it can be due to diminution of hemopoitic cell production, ineffective haemopoiesis or may be due to peripheral devastation of cells. The production of hemopoitic cell can be prejudiced in the bone marrow either by infections, toxins, and malignant cell infiltration leading to hypocellular marrow. Ineffectual hematopoiesis and dysplasia, maturation arrest of all the cell lines and peripheral sequestration of blood cells or peripheral destruction of all blood cells lineage can also be the cause of pancytopenia.^2^^-^^4^

Pancytopenia is a common hematological problem with an extensive differential diagnosis, and still the optimal diagnostic approach to pancytopenia remains undefined.^5^^-^^7^ Pancytopenia itself is not a disease but actually a triad of findings that may result from a number of disease processes associated with bone marrow both affecting it primarily or secondarily and resulting in pancytopenia.^8^ Most often the Pancytopenia is associated with organomegaly and lymphadenopathy usually suggests the possibility of malignancies or bone marrow failure syndromes but there are a number of other causes which can present in the similar way and are very easily treatable.^9^

Megaloblastic anemia has been known as a clinical entity for over a century, the first clinical case of pernicious anemia, which is one of the known causes of megaloblastic anemia, has been attributed to Thomas Addison in 1849.^10^ Megaloblastic anemia results from abnormal maturation of hematopoietic cells due to faulty DNA synthesis, the two vitamins namely vitamin B12 and folic acid are essential for DNA biosynthesis and the deficiency of either of these vitamin can result in asynchrony in the maturation of nucleus and cytoplasm of rapidly regenerating cells.^11^^,^^12^ Other than megaloblastic anemia, deficiency of these hematopoietic micronutrients in children has been incriminated to cause neuro-development dysfunction, abnormal movements and failure to thrive.^13^

Acquired aplastic anemia usually has an autoimmune basis that is diminution of the hematopoietic stem cells by direct toxicity mostly due to radiations, drugs, chemicals and viruses.^14^ Anemia of chronic disease is a mild to moderate anemia that occurs mostly in infections and inflammatory disorders^15^ and is frequently found in chronic kidney insufficiency, dialysis patients, congestive heart failure and renal transplantation.^16^

Anemia can also present in patients with newly diagnosed childhood acute lymphoblastic leukemia (ALL).^17^ Being the most common pediatric malignancies, ALL represents 25% of all childhood malignancies and approximately 75% of all cases of childhood leukemia.^18^

Bone marrow play a vital role in understanding the etiology of pancytopenia^19^, in time recognition of the underlying pathology will not only have an impact on the mortality and morbidity of the vulnerable pediatric patients but will also help us treat the most simple and easily treatable condition like megaloblastic anemia whose picture of presentation is very drastic but can be easily managed.

## METHODS

This was a retrospective descriptive study carried out at the Department of Pediatric of Rehman Medical Institute Peshawar from 01^st^ April to 15^th^ May, 2013and the data taken was of the last 6 years i.e. from 01 January 2006 – 31 December 2011.A total of 205 cases presenting with pancytopenia on peripheral blood smear were included in the study, fulfilling the inclusion and exclusion criteria.

The inclusion criteria for the study were, children in the age group 6 months to 14 years admitted in the hospital and who had pancytopenia on their peripheral blood smear. Pancytopenia was defined as hemoglobin (Hb) <10g%, absolute neutrophil count (ANC) <1.5*109/L, platelet count <100*109/L. The pancytopenia was labeled as severe if patient had two or more of the following: Hb <7 gm%, ANC <0.5*109/L, and platelet count <20*109/L.^20^

All those children who had received blood transfusion were excluded from the study. Detailed history was taken including dietary intake, ingestion of any drugs, worm’s infestation, and loss of blood and duration of onset of anemia.

The patient fulfilling the inclusion criteria at the time of admission had their complete blood count (including Hemoglobin, total Leukocytes count Differential leukocytes platelet count and ANC), Peripheral smear, serum vitamin B12 and bone marrow aspiration done. Bone marrow biopsy was done in cases where bone marrow aspiration was inconclusive. Ethical approval was obtained from the RMI Ethical Review Board regarding data collection and use for research purposes.

## RESULTS

Out of 14642 patients admitted to Department of Pediatrics, 205(1.4%) patients presented with pancytopenia. Out of 205 patients, 133 (64.87%) were males and 72 (35.13%) females, with male to female ratio of 1.84:1 (Table-I), their ages ranged from one month to 14 years. Maximum number of patients 87 (42.44%) were in the age group of 6 month to 5 years, followed by 72 (35.13%) in the 6 to 10 years age group while minimum number 46 (22.43%) were those exceeding 11 years of age (Table-I), all age group had a male predominance. The most common symptom was pallor in 170 (82.92%) cases and fever in 135 (62.85%) which was often prolonged for weeks, other symptoms included bruises, epistaxis, malena, petechial hemorrhages, hematuria and joint pains (Table-II)

Considering the etiological pattern of all the 205 cases that were included in the study due to pancytopenia, Aplastic anemia 58 (28.3%) was the most common cause of pancytopenia followed by leukemia 49 (23.9%) while megaloblastic anemia was found in 40 (19.51%) of cases followed by other less common problems like idiopathic thrombocytopenic purpura 16 (7.80%) iron deficiency anemia 9 (4.4%), Visceral leishmaniasis 6 (2.93%), anemia of chronic disorder 4 (1.95%) and malaria was found in 5 (2.44%) of cases (Table-III).

**Table-I:** Distribution of patient according to Gender and Age.

*Age*	*Male (n-133)*	*Female (n-72)*	*Total (n-205)*
6 months – 5 years	57	30	87 (42.44%)
6 years – 10 years	46	26	72 (35.12%)
11 years – 14 years	30	16	46 (22.44%)
Total	133	72	205 (100%)

**Table-II:** Etiological Pattern of Pancytopenia.

*Etiology*	*No. of cases (n-205)*	*Percentage*
Aplastic anemia	58	28.3
Leukemia’s	49	23.9
Megaloblastic Anemia	40	19.51
Idiopathic thrombocytopenia purpura	16	7.80
Iron deficiency anemia	9	4.4
Visceral leishmaniasis	6	2.93
Anemia of chronic disorder	4	1.95
Hypersplenism	5	2.44
Malaria	5	2.44
Hemolytic anemia	7	3.41
Myelodisplastic syndrome	3	1.46
Gaucher disease	1	0.49
Niemen pick disease	2	0.97

**Table-III:** Clinical feature of pancytopenia at presentation (n=205).

*Clinical Feature*	*No. of cases (n-205)*	*Percentage*
Pallor	170	82.92
Fever	135	62.85
Bruises	128	62.44
Petechial hemorrhage	40	19.51
Malena	30	14.63
Hematemesis	20	9.75
Joint/leg pain	60	29.27
Bleeding from gums	50	24.39
Epistaxis	60	29.27
Hematuria	25	12.19

## DISCUSSION

Pancytopenia is condition in which there is reduction of all the 3 peripheral blood lineage, it’s not an uncommon hematological problem countered in clinical practice and must be suspected on clinical grounds when a patient presents with unexplained pallor. In our study the frequency of Pancytopenia was 1.4%, this frequency in other studies is quite variable. According to a study conducted in Peshawar in 2000, they reported it to be 0.8%^21^, while a study done by Shazia Memon in Jomshoro reported it to be 3.57%^22^, while Adil et al^23^ and Kanchanalak et al^24^ reported it to be 12.6% and 1.2% respectively.

In our study male dominated female in all the age group with male to female ratio of 1.8:1, other studies done also had the some finding with male dominating female, a study from Peshawar done by Afzal Khan^25^ reported the male to female ratio of 1.5:1, while a study done by Amieleena C et al^9^ and Goel RG et al^26^ reported the male to female ratio of 1.64:1 and 1.76:1 respectively.

In our study many disease entities other than malignancies, significantly Aplastic anemia, Megaloblastic anemia and Infectious causes emerged as recognizable cause of pancytopenia. Aplastic anemia was the most common cause of pancytopenia diagnosed on bone marrow accounting for 58 (28.3%) of cases. Though aplastic anemia has a pattern of geographical variation opposite to that of leukemia, with higher incidence in the developing countries than the developed one.^27^ The studies done in European countries indicate the annual incidence of 2 new cases per million of population.^28^ Studies done in China^29^ and Thailand^30^ reported the frequency of aplastic anemia to be three folds to that of the western countries of the world. The studies done nationally reported, Afzal Khan^25^ from Peshawar, reported the aplastic anemia to be the most common disorder with a frequency of 40 (20.2%) cases out of 198 cases that were included in the study while Shazia Memon^22^ from Hyderabad reported the incidence of Aplastic anemia in 55 (23.9%) cases out of 230 cases included in study.

Another common etiological factor was nutritional deficiency anemia, especially megaloblastic anemia. It accounted for 40 (19.51%) patients presenting with pancytopenia. Megaloblastic anemia due to vitamin B12 deficiency is well recognizable cause of pancytopenia. Various study reported the range of pancytopenia in megaloblastic anemia to be varying from 11-47%.^8^ A study done in India by Bhatanger^31^, including 109 children in study presenting as pancytopenia the incidence of megaloblastic anemia was 28.4%. Study of 200 cases by Khunger^32^ reported megaloblastic anemia in 72% of cases. Other studies done nationally and internationally reported the incidence of megaloblastic anemia to be, Shazia et al^22^ including 230 children in the study reported the incidence of megaloblastic anemia in 40 (17.39%) cases. 11% in a study done be Gomber et al^33^ while it has reported to be 47% in a study done by Mubarik et al^34^ while Sarode et al^35^ from India reported the incidence of 80.5%.

In our study the incidence of Hematological malignancies was 49 (23.9%) cases, other studies done reported the incidence of hematological malignancies. In our study ALL 24 (11.7%) was the most common malignant condition presenting as Pancytopenia followed by AML 16 (7.80%) and the least common was CML 9 (4.4%), this finding of our study is almost in consistent with a study done including 230 cases^22^ of which 40 cases presenting with pancytopenia were malignant condition, the ALL 20 (8.69%) was the most common hematological malignancy presenting as pancytopenia followed by AML 5(2.17%) and CML 2 (0.89%). Afzal Khan from Peshawar reported the malignant condition to be one of the most common cause presenting as pancytopenia reporting it in 11.3% of cases^25^, other study done in Islamabad reported the similar results while a study done in USA reported the incidence of hematological malignancy in 4.5% cases per 100,000 children.

Idiopathic thrombocytopenic purpura accounted for 16(7.8%) of cases, being one of the common hematological problem in our study. It is also one of the commonest cause of purpura. Other studies^36^ have shown 32% to 48% frequency. While study done in Peshawar^25^ by Afzal Khan, reported it to be 15.7%.

## CONCLUSION

Pancytopenia is one of the importance occurrences in pediatric patients. Acute leukemia and bone marrow failure are the most common causes yet megaloblastic anemia and infections are the treatable and reversible causes of pancytopenia.

## Notes

***Source of funding:*** Dr. Anwar Zeb Jan.
